# Comparison of the Axes and Positions of the Uterus and Vagina Between Women With and Without Pelvic Floor Organ Prolapse

**DOI:** 10.3389/fsurg.2022.760723

**Published:** 2022-02-10

**Authors:** Song Li, Xuewei Wen, Zhenhua Gao, Kunbin Ke, Jing Yang, Haifeng Wang, Yin Mo, Yizhen Zeng, Yuan Li, Daoming Tian, Jihong Shen

**Affiliations:** ^1^Urology Department, Kunming Medical University First Affiliated Hospital, Kunming, China; ^2^Ophthalmology Department, Kunming Medical University First Affiliated Hospital, Kunming, China; ^3^Medical Imaging Department, Kunming Medical University First Affiliated Hospital, Kunming, China

**Keywords:** uterine axis, vaginal axis, uterine angle, vaginal angle, pelvic organ prolapse

## Abstract

**Purpose:**

To analyze the role of the axial positions of the uterus and vagina in providing pelvic floor support, encourage evaluations of pelvic floor function, and improve the understanding of the pathogenesis of pelvic organ prolapse.

**Methods:**

The lengths and angles of the upper, middle, and lower axes of the vagina, uterine body, and cervix of 81 women with prolapse (prolapse group) and 57 women without prolapse (non-prolapse group) were measured and compared using magnetic resonance images. The pelvic inclination correction system (PICS) line was also compared between the groups. The coordinate parameters of the anatomical points of the uterus and vagina were measured, and their positions were analyzed.

**Results:**

In the prolapse group, the uterine body-cervical angle, cervical-upper vaginal angle, uterine body-PICS line angle, cervical-PICS line angle, and lower vaginal-PICS line angle were smaller (*p* < 0.05) and the middle-lower vaginal angle, upper vaginal-PICS line angle, and middle vaginal-PICS line angles were larger (*p* < 0.05) than those in the non-prolapse group. The cervical length was longer (*p* < 0.05) and the middle and lower vaginal lengths were shorter (*p* < 0.05) in the prolapse group. The coordinate system revealed that the uterine and vaginal axes were shifted backward and downward in the prolapse group.

**Conclusion:**

Patients in the prolapse group were more likely to have retroversion and retroflexion of the uterus than those in the non-prolapse group. The vagina was shortened, turned forward, and straightened, and the uterus and vagina were shifted backward and downward in the prolapse group. Changes in the axial position of the uterus and vagina are important mechanisms of pelvic floor organ prolapse.

## Introduction

Female pelvic floor dysfunction is a chronic disease characterized by weakened supporting structures and presents as pelvic organ prolapse (POP), urinary incontinence, and sexual dysfunction. The incidence of POP has gradually increased. Fifty percent of parturient women experience POP-related symptoms, and 30–50% of adult women are affected by POP, including 11–19% who require surgery (1, 2). The reoperation rate is as high as 30% (3). Although POP is closely related to damage of the uterine and vaginal supporting structures and uterine and vaginal morphological changes, the pathogenesis of POP is unclear (4).

Pelvic floor organ prolapse is closely related to axial and mechanical balance disorders of the uterus and vagina; therefore, it was hypothesized that the axis, position, and shape of the uterus and vagina play an important role in maintaining the function of the pelvic floor organs. Few studies have reported the anatomical axes and positions of the uterus and vagina. Barnhart et al. (5) used magnetic resonance imaging (MRI) to measure the baseline dimensions of the undilated vagina in 28 women. Luo et al. (6) proposed a technique for quantifying the individual variability of the vaginal shape, axis, and size in healthy women. However, there are few comparative studies regarding the axes, shapes, and positions of the uterus and vagina in women with and without prolapse. In this study, differences in the axes, angles, shapes, and positions of the uterus and vagina on sagittal MR images were compared in women with and without POP to determine the diagnostic value of the shapes and positions of the uterus and vagina in patients with POP.

## Materials and Methods

Patients with POP who were clinically diagnosed *via* pelvic organ prolapse quantification (POP-Q) examinations at Kunming Medical University First Affiliated Hospital between September 2018 and November 2020 (*n* = 81; prolapse group) were included in this study. All patients in the prolapse group had at least one point of the vaginal wall or cervix one centimeter below the hymen. Fifty-seven volunteers of similar age and parity to patients in the prolapse group were included in the non-prolapse group. In the patients in the non-prolapse group, all points of the vaginal wall and cervix were at least one centimeter above the hymen on POP-Q examination. Sagittal MR images of the pelvis were obtained in all participants. Participants with contraindications to MRI were excluded from the study. This study was approved by the ethics committee of Kunming Medical University First Affiliated Hospital, and all participants provided written informed consent.

All participants underwent supine multi-plane proton density MRI with a 3T superconducting magnet (Philips Medical Systems Inc., WI, USA). T2-weighted fast recovery fast spin-echo MRIs in the axial, sagittal, and coronal planes were obtained using the following parameters: repetition time/time to echo, 3000/100–110; field of view, 26–28 cm; slice thickness, 3.0 mm interleaved; gap, 0.4 mm; scan time, 10 min; and 60 continuous images.

**Figure 1:**
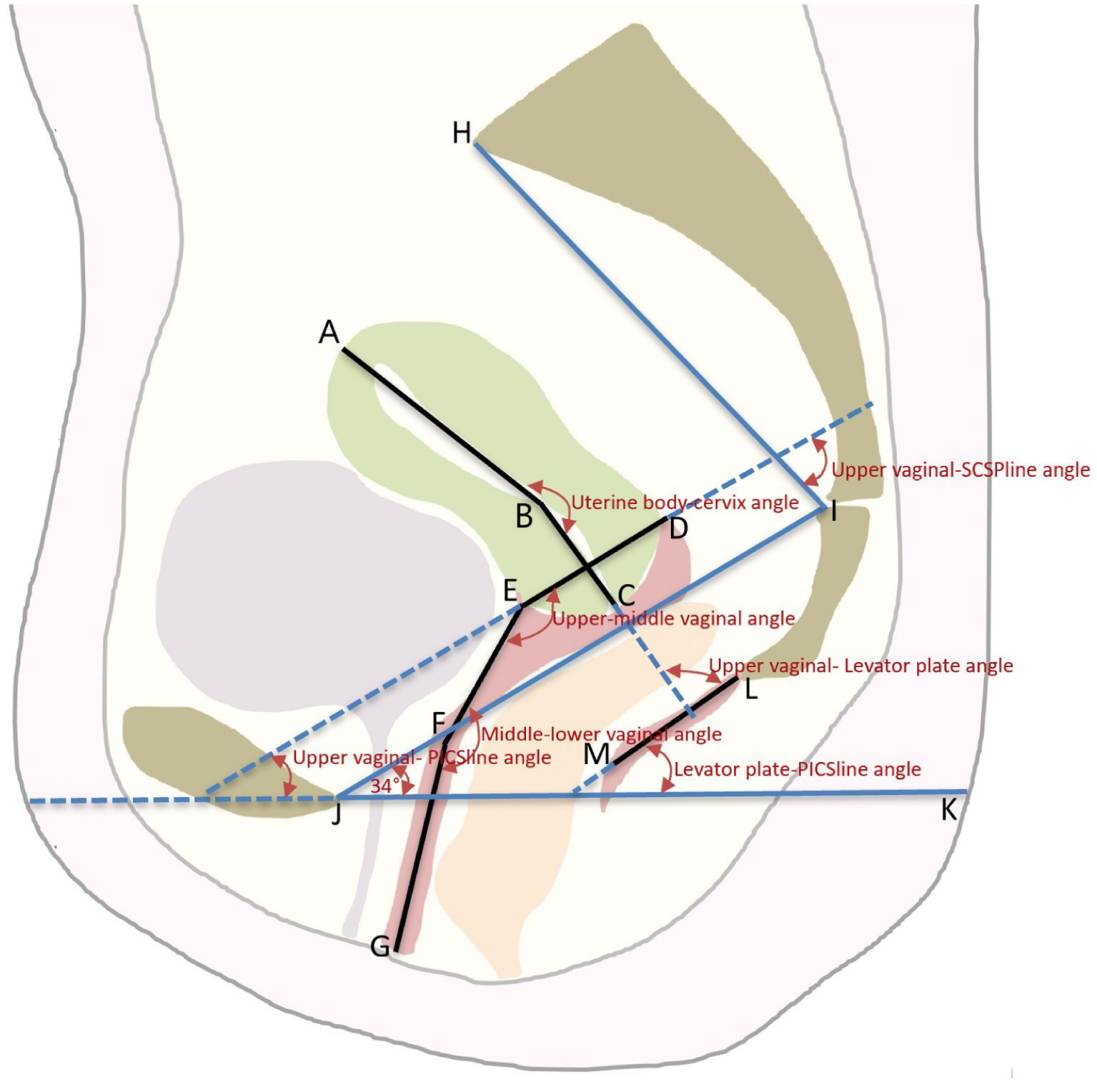
The schematic diagram of the axis and position of the Uterus and Vagina. An illustration of the pelvic landmarks, uterine and vaginal regions, uterine and vaginal axes, and uterine and vaginal angles used in this study is shown. A, uterine floor; B, internal orifice of the cervix; C, external orifice of the cervix; D, posterior vaginal fornix; E, anterior vaginal fornix; F, junction of the middle and lower vagina; G, vaginal introitus; AB, uterine body axis; BC, cervical axis; DE, upper vaginal axis; EF, middle vaginal axis; FG, lower vaginal axis; LM, levator plate; JI, SCIPP sacrococcygeal-inferior pubic point line; JK, pelvic inclination correction system (PICS) line; HI, SCSP line, obtained by connecting a line drawn from the sacral promontory to the junction of the fifth sacral and first coccygeal bone.

The DICOM MR images were imported into MIMICS version 19.01 software (Materialise Inc., Leuven, Belgium). Two radiologists with at least 5 years of experience in pelvic MRI diagnostics used a double-blind method to evaluate the uterine and vaginal morphology shown on the sagittal MR images at the same workstation. Seven points were used to determine the location and shape of the uterine and vaginal axes using the median sagittal plane of the MR image (Figure 1). The images marked by the two radiologists were then marked and examined by senior urologists.

**Figure 2:**
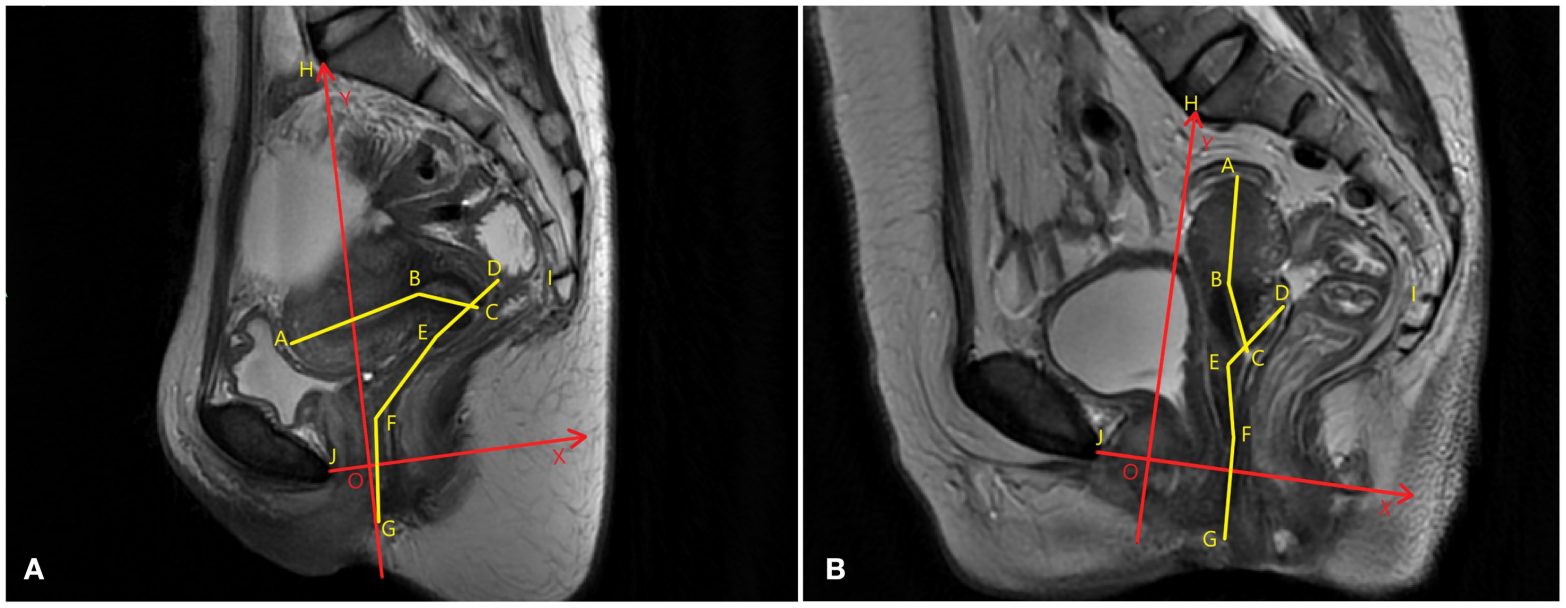
Uterine and vaginal analysis. **(A)** The central sagittal magnetic resonance (MR) image of a participant without prolapse is shown. **(B)** The sagittal median MR image of a participant with prolapse is shown. The sagittal plane analysis system of the axes, angles, and positions of uterus and vagina are established. To compare the shapes, positions, and angles of the uterus and vagina between different participants, a local coordinate system (XOY, in red) that quantifies the morphology was created. The OX axis was created by rotating the sacrococcygeal-inferior pubic point (SCIPP) line 34° clockwise. The OY axis is perpendicular to the X-axis through the sacral promontory, and the same coordinate system is used to compare the spatial position of each participant's uterus and vaginal axes.

The same analysis techniques were used to examine the sagittal plane MR images in all participants. The sagittal plane images were obtained with the participants in the supine position. The participants were asked to breathe calmly during the imaging. To quantify and measure the uterine vaginal axis, the uterus was divided into the uterine body and cervix. The vagina was divided into upper, middle, and lower segments. The upper vagina was analyzed using a line connecting the apex of the anterior and posterior fornix. The middle vagina was the area of the vagina superior to the pelvic septum, and the lower vagina was the area of the vagina inferior to the pelvic septum. The cervical axis was defined as the cervical canal connecting the internal orifice of the cervix with the external orifice of the cervix. The uterine body axis was defined as the line between the internal orifice of the cervix and the farthest point of the uterine floor passing through the uterine cavity. The uterine body-cervical angle was defined as the clockwise angle between the uterine body axis and the cervical axis. The angle between the uterine and vaginal axes and the angles between the uterine and vaginal axes and the pelvic inclination correction system (PICS) line were calculated. The line from the sacral promontory to the junction of the fifth sacral and first coccygeal bone was termed the SCSP line. The SCSP line was determined using the median sagittal MR image. The angles between the uterine and vaginal axes and the SCSP line were measured to evaluate the angular relationships between the uterine and vaginal axes and the sacrum. The angles and lengths of the uterine and vaginal axes were measured using MIMICS software. A local coordinate system was created by combining the bony mark H (Sacral promontory) and the PICS line (6, 7). The PICS line served as the x-axis and was established by rotating the sacrococcygeal-inferior pubic point (SCIPP) 34° clockwise. The y-axis was parallel to the gravity line, and the same coordinate system was used to compare the shape and position of each participant's uterus and vagina (Figure 2). The y-axis connected the sacral promontory to the X.

The participants' demographic data, including age, height, weight, and body mass index (BMI), were compared between the two groups. Continuous variables were compared using the independent-sample *t*-test (parametric variables) and the Mann-Whitney U test (nonparametric variables). All *p*-values were two-sided, and statistical significance was set at *p* < 0.05. Confidence intervals were set at 95%. All statistical analyses were performed using SPSS software (version 22.0; IBM Corp., Armonk, NY, USA) and MedCalc software (version 15.2.2; MedCalc Software, Ostend, Belgium).

## Results

There were no significant differences in patient demographics between the two groups (Table 1). In the prolapse group, 39 patients had a cystocele, 30 had uterine prolapse, and 12 had a rectocele. Among the 39 participants with a cystocele, 22 also had uterine prolapse and five also had a rectocele. Four patients had both a rectocele and uterine prolapse. Nine patients had stage I prolapse, 21 had stage II prolapse, 22 had stage III prolapse, and 29 had stage IV prolapse.

**Table 1:** Demographic data.

**Variable**	**Non-prolapse group (mean ±SD) (*n* = 57)**	**Prolapse group (mean ±SD) (*n* = 81)**	*P*-value^*^
Age (years)	60.55 ± 11.14	61.15 ± 11.82	0.761
Height (cm)	165.23 ± 18.71	162.65 ± 22.92	0.469
Weight (kg)	54.13 ± 12.12	56.45 ± 10.23	0.240
BMI (kg/m^2^)	23.66 ± 2.51	24.40 ± 2.97	0.131
Vaginal parity	2.90 ± 1.67	3.07 ± 1.73	0.566

* *P-values based on an independent-sample T-test. BMI, bone mineral density; SD, standard deviation*.

### Measurement of the Uterine and Vaginal Angles

The uterine body-cervical angle (*p* < 0.001), cervix-upper vaginal angle (*p* < 0.001), uterine body-PICS line angle (*p* < 0.001), and cervical-PICS line angle (*p* < 0.003) were smaller in the prolapse group than in the non-prolapse group.

As shown in Table 2, the middle vaginal-lower vaginal angle was greater in the prolapse group than in the non-prolapse group (*p* < 0.001). The upper vaginal-PICS line (*p* < 0.001) and middle vaginal-PICS line (*p* < 0.001) angles were greater in the prolapse group than in the non-prolapse group. The lower vaginal-PICS line (*p* < 0.048) and lower vaginal-levator plate (*p* < 0.001) angles were smaller in the prolapse group than in the non-prolapse group. The levator plate-PICS line angle was greater in the prolapse group than in the non-prolapse group (*p* < 0.001).

**Table 2:** Measurement of the uterus and vaginal angles.

**Variable (degrees)**	**Non-prolapse group (mean ±SD) (*n* = 57)**	**Prolapse group (mean ±SD) (*n* = 81)**	***P*-value**
Uterine body-cervix angle	219.97 ± 30.45	162.48 ± 45.24	<0.001^#^
Cervix-upper vaginal angle	261.14 ± 18.71	235.77 ± 22.92	<0.001^*^
Upper-middle vaginal angle	147.61 ± 14.75	142.88 ± 22.25	0.751^#^
Middle-lower vaginal angle	158.94 ± 9.03	180.52 ± 15.24	<0.001^#^
Uterine body-SCSP line angle	195.93 ± 35.44	125.81 ± 41.23	<0.001^#^
Cervix- SCSP line angle	157.19 ± 24.79	143.70 ± 20.61	0.003^#^
Upper vaginal-SCSP line angle	75.65 ± 10.78	88.98 ±16.64	<0.001^#^
Middle vaginal-SCSP line angle	109.12 ± 10.01	126.82 ± 17.41	<0.001^#^
lower vaginal-SCSP line angle	130.49 ± 8.26	126.10 ± 13.87	0.006^#^
Uterine body-PICS line angle	151.77 ±3 5.48	82.71 ± 40.80	<0.001^#^
Cervix-PICS line angle	112.83 ± 26.95	100.78 ± 19.87	0.003^*^
Upper vaginal-PICS line angle	31.26 ± 10.31	46.12 ± 17.16	<0.001^#^
Middle vaginal-PICS line angle	65.07 ± 10.17	83.69 ± 17.68	<0.001^#^
Lower vaginal-PICS line angle	86.21 ± 6.64	83.53 ± 14.35	0.048^#^
Levator plate-PICS line angle	28.59 ± 8.41	45.20 ± 11.95	<0.001^#^
Uterine body-levator plate angle	124.14 ± 36.71	38.75 ± 40.31	<0.001^*^
Cervix-levator plate angle	84.57 ± 24.20	56.03 ± 22.46	<0.001^*^
Upper vaginal-levator plate angle	3.22 ± 12.44	1.47 ± 19.54	0.998^#^
Middle vaginal-levator plate angle	37.12 ± 10.88	39.02 ± 20.06	0.654^#^
Lower vaginal-levator plate angle	57.99 ± 9.76	38.49 ± 17.61	<0.001^#^

* *P-values obtained using the independent-sample T-test*.

# *P-values obtained using the Mann-Whitney U test*.

*SD, standard deviation; SCSP, line from the sacral promontory to the junction of the fifth sacral and first coccygeal bone; PICS, pelvic inclination correction system*.

### Measurements of the Length of the Uterus and Vagina

As shown in Table 3, the cervical length was longer in the prolapse group than in the non-prolapse group (*p* < 0.001). The lengths of the middle vagina (*p* < 0.001) and lower vagina (*p* < 0.001) were shorter in the prolapse group than in the non-prolapse group. The total length of the upper and middle vagina was shorter in the prolapse group than in the non-prolapse group (*p* < 0.001). The total vaginal length was shorter in the prolapse group than in the non-prolapse group (*p* < 0.001). The anterior and posterior diameters of the levator ani muscle hiatus increased (*p* < 0.001).

**Table 3:** Lengths of the uterus and vagina.

**Lengths (mm)**	**Non-prolapse group (mean ±SD) (*n* = 57)**	**Prolapse group (mean ±SD) (*n* = 81)**	***P*-value**
Uterine body	50.62 ± 13.91	46.21 ± 12.60	0.052^#^
Cervix	19.96 ± 4.94	29.71 ± 12.84	<0.001^#^
Uterine	70.58 ± 17.27	75.93 ± 21.87	0.232^#^
Upper vaginal	27.57 ± 5.70	27.85 ± 7.84	0.764^#^
Middle vaginal	29.52 ± 5.08	22.44 ± 5.47	<0.001^*^
Lower vaginal	29.81 ± 4.33	23.94 ± 5.86	<0.001^#^
Upper and middle vaginal	57.10 ± 9.13	50.30 ± 10.85	<0.001^#^
Total vaginal	86.92 ± 12.17	74.25 ± 14.59	<0.001^*^
Levator ani muscle hiatus	49.00 ± 6.06	58.43 ± 6.61	<0.001^*^

* *P-value obtained using the independent-sample T-test*.

# *P-value obtained using the Mann-Whitney U test*.

*SD, standard deviation*.

### Positions of the Uterus and Vagina

**Figure 3:**
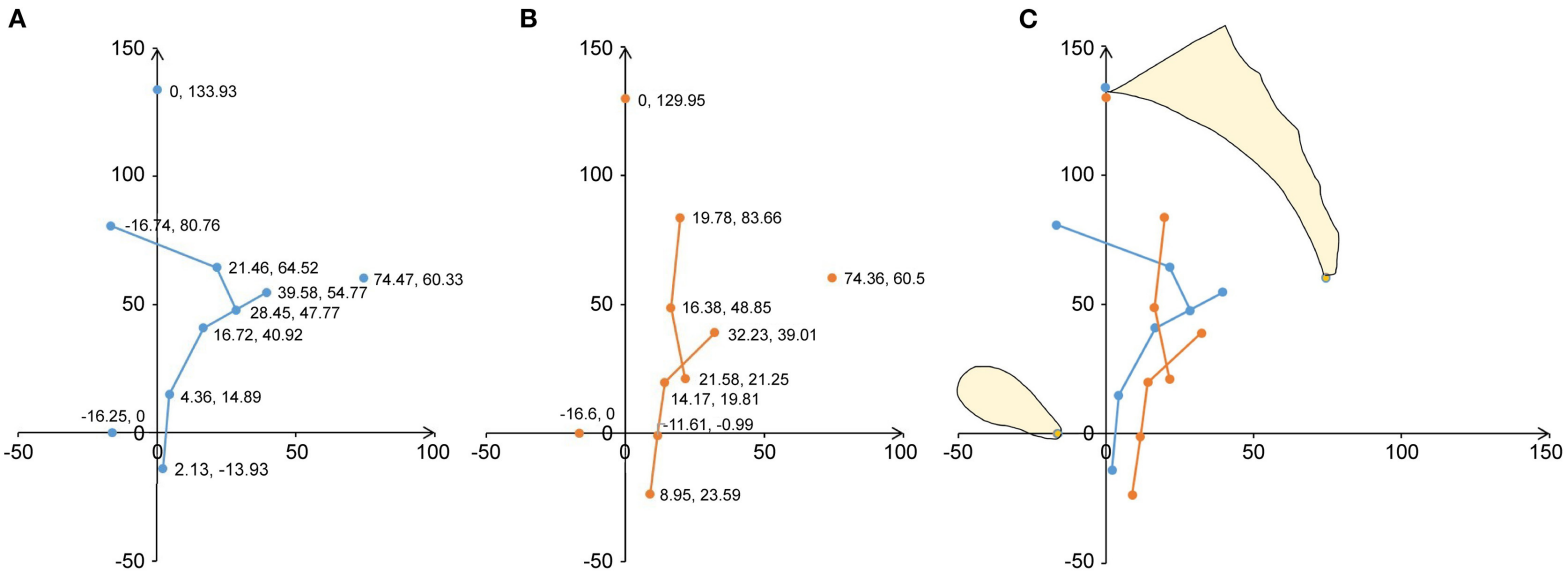
Analysis of the average shape and position of the geometric structure of the uterus and vagina on the median sagittal plane. A mid-sagittal shape and the mean shape of the uterine axis and vaginal axis are shown in terms of the local pelvic inclination correction system (PICS) coordinates. **(A)** The geometric results of 57 participants without prolapse are shown. The blue dots show the location and coordinate values of the average coordinates of the uterine and vaginal anatomical marks. The blue line indicates the average shape. **(B)** The geometric results of 81 patients with prolapse are shown. The orange dots show the position and coordinate values of the average coordinates of the uterine and vaginal anatomical marks. The orange line indicates the average shape. **(C)** The average coordinate positions and shapes of the uterine and vaginal anatomical markers for the prolapse and non-prolapse groups are shown. Orange is the average shape of the prolapse group and blue is the average shape of the non-prolapse group.

As shown in Figure 3 and Table 4, the uterine and vaginal axes, the external orifice of the cervix, the posterior fornix, the division of the middle and lower parts of the vagina, and the external orifice of the vagina of the prolapse group were shifted backward and downward compared to those of the non-prolapse group. The uterine floor was shifted backward in the prolapse group compared to that of the non-prolapse group. The internal cervical orifice and anterior fornix were shifted downward in the prolapse group compared to the non-prolapse group.

**Table 4:** Coordinate parameters of the uterus and vagina.

**Variable (mm)**	**Non-prolapse group (mean ±SD) (*n* = 57)**	**Prolapse group (mean ±SD) (*n* = 81)**	***P*-value**
A-x value	−16.74 ± 23.15	19.78 ± 27.73	<0.001^*^
A-y value	80.76 ± 22.94	83.66 ± 21.76	0.452^*^
B-x value	21.46 ± 12.65	16.38 ± 17.56	0.130^#^
B-y value	64.52 ± 9.96	48.85 ± 20.22	<0.001^#^
C-x value	28.45 ± 13.51	21.58 ± 16.40	0.010^*^
C-y value	47.77 ± 7.85	21.25 ± 26.54	<0.001^#^
D-x value	39.58 ± 14.14	32.23 ± 15.51	0.005^*^
D-y value	54.77 ± 9.14	39.01 ± 15.53	<0.001^#^
E-x value	16.72 ± 11.03	14.17 ± 14.56	0.302^#^
E-y value	40.92 ± 7.41	19.81 ± 15.13	<0.001^#^
F-x value	4.36 ± 9.91	11.61 ± 13.50	0.001^#^
F-y value	14.89 ± 6.34	−0.99 ± 13.99	<0.001^#^
G-x value	2.13 ± 11.00	8.95 ± 15.65	0.012^#^
G-y value	−13.93 ± 5.54	−23.59 ±14.36	<0.001^#^
H-x value	0	0	
H-y value	133.93 ±11.69	129.95 ± 11.48	0.049^*^
I-x value	74.47 ± 9.35	74.36 ± 11.69	0.954^*^
I-y value	60.33 ± 4.53	60.50 ± 5.23	0.845^*^
J-x value	−16.25 ± 8.65	−16.60 ± 12.02	0.852^*^
J-y value	0	0	

* *P-value obtained using the independent-sample T-test*.

# *P-value obtained using the Mann-Whitney U test*.

*SD, standard deviation; A, Uterine floor; B, Internal orifice of the cervix; C, External orifice of the cervix; D, posterior vaginal fornix; E, anterior vaginal fornix; F, The junction of the middle and lower vagina; G, vaginal introitus; H, Sacral promontory; I, the junction between the fifth sacral and first coccygeal bone; and J, the inferior point of the pubic symphysis*.

## Discussion

In this study, the uterine and vaginal axes of patients with prolapse were significantly different than those of women without prolapse. However, determining the axes and angles of the uterus and vagina during a gynecological examination is difficult. The use of a colposcope straightens the vagina, affecting the vaginal axis and angle (5). Postmortem muscle fixation and rectal dilatation cause forward movement and shortening of the vagina in cadavers, resulting in a large error between pre- and postmortem evaluations. The boundaries of the uterus and vagina are visible in sagittal MR images; therefore, the positions and axes of the uterus and vagina can be measured and quantified using MRI without distortion (3). MRI is useful to study the pelvic organs and the functional anatomy of the pelvic floor in living women.

The vaginas of participants without prolapse were located on an almost horizontal axis above the pelvic diaphragm with a forward and upper protuberance, and the axis of the upper part of the vagina pointed to the sacral canal. In healthy women, the position and angle of the vagina are not significantly affected by changes in body position (5, 8). The angle between the upper and middle vaginal horizontal lines was significantly larger in the prolapse group than in the non-prolapse group in this study. The angles of the uterus and vagina determined in this study provide a theoretical reference for pelvic floor surgeons. The vaginal angle of patients with prolapse differs from that of patients without prolapse. The changes in the axis and position of the vagina after POP must be well-understood when surgically repairing the prolapse. The axes and angles of the uterus and vagina may be restored *via* pelvic floor reconstruction, maintaining the dynamic balance between the pelvic floor organs.

Betschart et al. (7) developed the PICS to analyze pelvic MR images. The average vaginal axis was reported by Luo et al. as X. Lee et al. (9) reported that the middle part of the vagina is tilted forward and the angles between the various parts of the vagina are larger in women who underwent a hysterectomy than in women with an intact uterus. The angle between the upper and lower vaginal axes in the upright and supine positions has been reported as 130°, and the shape of the vagina has been reported as X (10, 11). Due to the relationship between the upper part of the vagina and the levator ani muscle, an increase in abdominal pressure results in a more horizontal position of the vagina, though the shape of the vagina does not change (12). Nichols et al. (13) reported an obvious division of the vagina into upper and lower parts at the levator ani muscle hiatus. The upper part of the vagina lies horizontally from the pelvic diaphragm to the cervix while the lower part lies vertically from the vaginal orifice to the pelvic diaphragm (5). The angles between the vaginal and PCL lines and between vaginal parts are significantly different in women with and without prolapse, and the lower vagina is more retroverted in patients with prolapse (14). Therefore, POP is closely related to axial disorders and a biomechanical imbalance between the uterus and vagina.

In patients with POP, the uterus was tilted and flexed backward toward the sacrum, and the angles between the different parts of the vagina were larger than those of women without prolapse. The vaginal axis shifted forward and became flatter as the vagina was shifted backward and downward, closer to the sacrum and coccyx, in patients with POP. Changes in the axes, angles, and positions of the uterus and vagina in patients with POP may increase the vulnerability of the uterus and vagina to abdominal pressure and gravity (15).

In patients without prolapse, the middle and upper vaginal axes are at sharp angles to the levator ani muscle plate. POP is closely related to defects of the pubic and coccyx junctions of the levator anus muscle (16). As intra-abdominal pressure increases, the levator ani muscle plate increases due to muscle contraction, becoming more horizontal and supporting the pelvic contents (17). In this study, patients with POP had a loss of the levator angle. The angle between the levator plate and the PICS line was greater in the prolapse group than in the non-prolapse group in this study, suggesting that the loss of the normal support of the levator muscle contributes to POP. Loss of the levator anus muscle plate angle may reflect the loss of the support of the uterine sacral ligament and levator anus muscle (18).

The middle and upper vaginal angles changed with the levator anus muscle plate angle in this study. The changes in the middle and upper vaginal axes were consistent with those of the levator anus muscle plate in both direction and degree. Singh et al. (19) evaluated the relationship between the vagina and the levator ani plate using the levator anus plate-vaginal angle and found that a return of the levator ani plate-vaginal angle to a normal or nearly normal value may be associated with the success of repair surgeries in patients with POP. The pathological extension of the hysterosacral and main ligaments causes the upper vagina to turn forward (11). The levator ani muscle plate can reduce the trend of vaginal reversal or prolapse (10).

The enlargement of the levator muscle hiatus in the prolapse group of this study resulted in a central hernia in the pelvic floor, which increases the risk of POP (19). POP is more likely to occur when the levator ani muscle plate axis is affected, and the upper vagina may be inverted and shifted forward when the main ligament and the hysterosacral ligament are pathologically elongated or relaxed. Restoration of the levator ani muscle plate axis *via* pelvic floor reconstruction and repairing the levator muscle angle may improve the functional recovery of patients with POP.

The results of this study confirm that the uterus is retroverted and flexed and that the cervix is longer, straighter, and shifted backward and downward in patients with POP. The vagina is shortened, straightened, and shifted forward in these patients. The uterus and vagina of women in the prolapse group were more likely to develop prolapse due to intra-abdominal pressure.

The total length of the vaginal axis, the lengths of the middle and lower vaginal axes, and the lengths of the middle and upper vaginal axes were shorter in the prolapse group than in the non-prolapse group in this study. In healthy women, the portion of the vagina superior to the levator ani muscle plate is parallel to the levator ani muscle plate and is long enough to not be affected by increased intra-abdominal pressure (11, 20). When intra-abdominal pressure increases, the vagina is lowered and shifted backward, squeezing the levator ani muscle. The reflex contraction of the levator ani muscle increases to support the cervix and upper vagina (19).

Vaginal shortening above the levator ani hiatus results in a loss of balance of the biomechanical relationship in patients with POP, and intra-abdominal pressure causes the shortened vaginal fornix to shift over the levator ani hiatus. Medina et al. (20) reported that vaginal length shortening is associated with POP recurrence. A short upper part of the vagina is more likely to prolapse than a long upper part (21). The exact location of the vaginal axis can be determined by extending the central vaginal axis to the sacrum. The upper part of the normal vagina lies on the axis pointing to the sacral depression (8), while the superior vaginal axis is located between the third and fourth sacral vertebrae with an average vector point just above the center of the fourth sacral vertebra (13).

The SCSP line is a novel reference line introduced in this study. The upper and middle vaginal-SCSP line angles were larger in the prolapse group than in the non-prolapse group in this study. The average vector point was significantly higher than the center of the fourth sacral vertebra. The change in the vaginal axis relative to the sacrum and coccyx may result in a loss of vaginal support, rendering the vagina more vulnerable to changes in abdominal pressure in patients with POP. The more severe the degree of prolapse, the higher the incidence of abnormal vaginal morphology (22–24). The loss of the vaginal angle is related to prolapse (18). Vaginal morphological changes can predict the degree of prolapse and indicate the type of prolapse (25). Huebner et al. (23, 24) reported a positive phase between vaginal distortion and the abnormal decline of pelvic organs on MRI, suggesting that vaginal distortion may indicate pelvic floor dysfunction (25, 26).

Maintenance of the shapes of the uterus and vagina depends on the support of the paravaginal structure of the uterus and the mechanical balance during increased intra-abdominal pressure. Relaxation or injury of the supporting structure or increased intra-abdominal pressure disrupts the biomechanical balance of the pelvic floor structure, resulting in changes of the axes, positions, and shapes of the uterus and vagina (4, 26, 27).

Proper biomechanical balance is needed to avoid POP. The results of this study indicate that the vaginal axis of patients with POP shifted forward and that the mechanical directions of the uterus and the vaginal axes changed during increased intra-abdominal pressure. The force was distributed to the vaginal orifice through the vaginal wall instead of the sacrum, coccyx, and levator ani, leading to POP.

This study is not without limitations. First, the MR images were obtained with participants in the supine position, which offsets the effects of gravity. Second, the actual three-dimensional shapes and structures of the pelvic organs are more complex than those observed using two-dimensional images. Three-dimensional reconstructions of pelvic floor organs and a three-dimensional coordinate system should be used to determine the axes, shapes, and positions of the uterus and vagina in future studies.

Anatomical repair is of great significance in pelvic floor reconstruction surgery. Using the data obtained in this study, a clinical predictive model can be constructed to predict the incidence of POP and analyze the mechanism of POP, which will be helpful when planning pelvic floor reconstruction surgeries. Pelvic floor reconstructions that restore the axes, shapes, and positions of the uterus and vagina will reduce the incidence of recurrent prolapse and complications (12, 27, 28). Therefore, the results of this study provide valuable information regarding pelvic floor support and will play a role in the improvement of pelvic floor reconstruction surgery and early evaluation and prediction methods for POP.

## Data Availability Statement

The raw data supporting the conclusions of this article will be made available by the authors, without undue reservation.

## Ethics Statement

The studies involving human participants were reviewed and approved by Kunming Medical University First Affiliated Hospital. The patients/participants provided their written informed consent to participate in this study.

## Author Contributions

SL, XW, and JS contributed to conception and design of the study. ZG, KK, and JY organized the database. HW and YM performed the statistical analysis. SL and XW wrote the first draft of the manuscript. DT, YL, and YZ wrote sections of the manuscript. All authors contributed to manuscript revision, read, and approved the submitted version.

## Funding

This study was supported by the Scientific Research Foundation Project of the Department of Education of Yunnan Province [Grant Number: 2020J0167] and the Scientific and Technological Innovation Team for Research and Application of Female Pelvic Floor Dysfunction in Colleges and Universities in Yunnan Province [Grant Number: K1322112].

## Conflict of Interest

The authors declare that the research was conducted in the absence of any commercial or financial relationships that could be construed as a potential conflict of interest.

## Publisher's Note

All claims expressed in this article are solely those of the authors and do not necessarily represent those of their affiliated organizations, or those of the publisher, the editors and the reviewers. Any product that may be evaluated in this article, or claim that may be made by its manufacturer, is not guaranteed or endorsed by the publisher.
